# Isolation of a Potential Probiotic *Levilactobacillus brevis* and Evaluation of Its Exopolysaccharide for Antioxidant and α-Glucosidase Inhibitory Activities

**DOI:** 10.4014/jmb.2304.04043

**Published:** 2023-10-13

**Authors:** Se-Young Kwun, Jeong-Ah Yoon, Ga-Yeon Kim, Young-Woo Bae, Eun-Hee Park, Myoung-Dong Kim

**Affiliations:** 1 Department of Food Biotechnology and Environmental Science, Kangwon National University, Chuncheon 24341, Republic of Korea; 2 Department of Food Science and Biotechnology, Kangwon National University, Chuncheon 24341, Republic of Korea; 3 Institute of Fermentation and Brewing, Kangwon National University, Chuncheon 24341, Republic of Korea

**Keywords:** *Levilactobacillus brevis*, probiotic, exopolysaccharide, antioxidant activity, α-glucosidase inhibitory activity

## Abstract

The probiotic properties of ten lactic acid bacteria and antioxidant and α-glucosidase inhibitory activities of the exopolysaccharide (EPS) of the selected strain were investigated in this study. *Levilactobacillus brevis* L010 was one of the most active strains across all the in *vitro* tests. The cell-free supernatant (50 g/l) of *L. brevis* L010 showed high levels of both α-glucosidase inhibitory activity (98.73 ± 1.32%) and 2-diphenyl^-1^-picrylhydrazyl (DPPH) radical-scavenging activity (32.29 ± 3.86%). The EPS isolated from cell-free supernatant of *L. brevis* L010 showed 2,2'-azino-bis (3-ethylbenzothiazoline-6-sulfonic acid) radical-scavenging activity (80.27 ± 2.51%) at 80 g/l, DPPH radical-scavenging activity (38.19 ± 9.61%) at 40 g/l, and ferric reducing antioxidant power (17.35 ± 0.20 mg/l) at 80 g/l. Further, EPS exhibited inhibitory activities against α-glucosidase at different substrate concentrations. Kinetic analysis suggests that the mode of inhibition was competitive, with a kinetic constant of *K*_m_ = 2.87 ± 0.88 mM and *V*_max_ = 0.39 ± 0.06 μmole/min. It was concluded that the EPS might be one of the plausible candidates for possible antioxidant and α-glucosidase activities of the *L. brevis* L010 strain.

## Introduction

Probiotics provide health benefits to the host when administered in appropriate amounts [1]. Lactic acid bacteria (LAB) represent probiotic strains often found in fruits, dairy, and fermented foods such as yogurt, kimchi (Korean traditional fermented cabbage), pickles, and makgeolli (Korean rice wine) [2-5]. Commonly used LAB probiotics include the species of *Leuconostoc*, *Levilactobacillus*, *Bifidobacterium*, and *Weissella* [5-7].

Lee and Kim [8] reported that *Leuconostoc mesenteroides* MKSR possesses α-glucosidase inhibitory activity and cholesterol-lowering effects. Treatment of *Latilactobacillus sakei* OK67 ameliorated high-fat diet–induced blood glucose intolerance [9]. In addition, *Levilactobacillus brevis* KU15006 exhibited antimicrobial activity against foodborne pathogens and anti-diabetic properties [10]. For a potential probiotic to be able to exert its beneficial effects, it must be able to survive in the gastrointestinal environment. Therefore, the essential characteristics for a LAB to be functional as a probiotic include the following: (i) tolerance to low pH and bile salt stress conditions, (ii) ability to adhesion to intestinal surfaces, and (iii) inhibitory activity against pathogenic bacteria [11]. *Lacticaseibacillus rhamnosus* GG (LGG) is a classic example of a probiotic strain with all three characteristics: acid and bile tolerance and excellent adhesion to human tissue. LGG has been used as the control in many studies with which other *Lactobacillus* spp. were compared [12-14].

Type 2 diabetes mellitus (T2DM) is characterized by hyperglycemia and impaired carbohydrate metabolism. One of the therapeutic strategies for T2DM is inhibiting carbohydrate degradation by α-glucosidase [15, 16]. Acarbose is an α-glucosidase inhibitor used to treat diabetes; however, this drug has side effects such as flatulence, abdominal pain, and diarrhea [16, 17]. Recent studies have identified LAB that inhibit α-glucosidase activity and function as acarbose substitutes. *Bifidobacterium bifidum* F-35 and *Lactiplantibacillus plantarum* IF2-14 exhibited inhibitory activity against α-glucosidase [14, 18].

Chen *et al*. [14] examined antioxidant and α-glucosidase inhibitory activities to identify potential anti-diabetic and probiotic LAB. *L. rhamnosus* Z7 showed significantly high antioxidant and α-glucosidase activities. A recent study has suggested that exopolysaccharides (EPS) might result in inhibitory activity [19].

Ten lactic acid bacteria were screened for their probiotic properties in this study. The antioxidant and α-glucosidase inhibitory activities of the EPS of selected strains were investigated.

## Materials and Methods

### LAB Strains

Ten LAB strains isolated from Korean fermented foods [20] were used in this study and are shown in Table 1.

### Preparation of Cell Lysates and Cell-Free Supernatant

LAB strains were incubated in MRS broth (MB Cell, Korea) at 30°C for 48 h and centrifuged at 10,000 ×*g* for 1 min. A cell-free supernatant was obtained by filtering the supernatant using a membrane (0.22 μm, Sartorius, Germany). Cells were washed three times with phosphate-buffered saline (PBS, pH 7.4; Welgene, Korea), disrupted using an ultrasonic homogenizer (Sonics, USA), and centrifuged at 10,000 ×*g* for 1 min to obtain clear cell lysates.

### α-Glucosidase Inhibition Assay

Inhibition of α-glucosidase was determined following a previously described method [14] with minor modifications. The cell-free supernatant or cell lysates (50 μl) were mixed with 50 μl of α-glucosidase (1.0 U/ml, Sigma-Aldrich, USA). After incubation at 37°C for 5 min, 50 μl of 5 mM *p*-nitrophenyl-α-D-glucopyranoside (*p*-NPG; Sigma-Aldrich) was added. The reaction proceeded at 37°C for 30 min and was terminated by adding 100 μl of 0.1 M sodium carbonate. Inhibitory activity was determined by measuring the absorbance at 405 nm to determine the amount of *p*-nitrophenol released from *p*-NPG [14].

### DPPH Radical-Scavenging Activity Assay

The cell lysates or cell-free supernatant (20 μl) were mixed with 180 μl of 2,2-Diphenyl^-1^-picrylhydrazyl (DPPH) radical solution (0.4 M) and incubated in the dark at 25°C for 30 min. After centrifugation at 10,000 ×*g* for 1 min, the absorbance was measured at 517 nm. The DPPH free radical-scavenging activity was determined as previously described [21].

### Adhesion to Caco-2 Cells

Caco-2 cells (Korea Cell Line Bank, Korea) were cultured according to a previously described method [22]; LGG (KCTC5033; Korean Collection for Type Cultures, Korea) was used as a positive control. The Caco-2 cells were inoculated at a cell density of 1.0 × 10^5^ cells/ml in a six-well plate and maintained for two weeks. The LAB were inoculated into Caco-2 cell-containing medium at a concentration of 1.0 × 10^8^ CFU/ml following incubation at 37°C for 2 h. After removing unbound LAB by washing three times with PBS, adhered LAB was released by treating with Triton X-100 (0.05%, Sigma-Aldrich). The mixture of Caco-2 cells and LAB was plated on MRS agar using the pour plate method [23] and incubated at 30°C for 2 days. The adhesion rate was estimated using a previously reported equation [12].

### Tolerance to Artificial Gastric Juice and Bile Juice

The tolerance of the strains to artificial gastric juice and bile juice was determined according to a previously described method [24] with minor modifications. LAB growing exponentially in MRS were harvested when the absorbance at 600 nm (OD_600_) reached 1.0, and 50 μl of culture was inoculated into a sterile 96-well plate. Subsequently, 150 μl of artificial gastric juice (pH 2.5, adjusted using pepsin) or bile juice (1% ox gall in MRS) was added. After incubation at 30°C for 2 h, cell growth was determined by measuring the OD_600_.

### Antimicrobial Activity Assay

The disk diffusion method [25] was used to determine antimicrobial activity against foodborne pathogens (*Bacillus cereus* KCTC1012, *Escherichia coli* O157: H7 KCCM40406, and *Staphylococcus aureus* KCTC1916). Pathogens growing exponentially (OD_600_ = 0.8–1.0) in nutrient broth (MB cell) were spread onto nutrient agar. A paper disk was placed on the surface of the agar, and 10 μl of LAB showing exponential growth in MRS was loaded on each paper disk. The plates were incubated at 37°C for 24 h. Ampicillin (50 mg/l, Sigma-Aldrich) and LGG were positive controls.

### Identification of LAB

Genomic DNA was extracted as previously described [26]. The 16S rRNA gene sequences were compared using BLAST (National Center for Biotechnology Information, USA). A phylogenetic tree of the retrieved sequences was constructed using the neighbor-joining method in MEGA 11.0 software (version 11.0.8, USA) [27].

### Determination of Specific Growth Rate of *L. brevis* L010

The specific growth rate of *L. brevis* L010 was determined under various culture temperatures (25, 30, 37, 40, and 45°C) and medium acidity (pH 4, 5, 6, 7, and 8). The culture medium acidity was adjusted using a buffer (pH 4 and 5: citrate buffer; pH 6 and 7: potassium phosphate buffer; pH 8: Tris-HCl buffer). *L. brevis* L010 pre-cultured in MRS at 30°C for 12 h was inoculated into 200 ml MRS broth at an initial OD_600_ of 0.1 and then incubated in shake flasks (200 rpm). The specific growth rate (h^-1^) was determined during the exponential growth phase [28].

### EPS Extraction

EPS was extracted using a previously described method [29]. *L. brevis* L010 cultured at 30°C for 48 h was boiled for 10 min to inactivate enzymes following trichloroacetic acid addition at a final concentration of 10%. The mixture was incubated at 4°C for 4 h, and the precipitate was removed via centrifugation (9,000 ×*g*) for 10 min. Two volumes of ice-cold ethanol were added to the supernatant, and the mixture was stored at 4°C for 12 h. EPS was precipitated via centrifugation at 9,000 ×*g* for 20 min, dissolved in sterile water, dialyzed against water using a dialysis tube (molecular weight cut-off of 3,500 Da; Thermo Fisher Scientific., USA), and then lyophilized.

### Antioxidant Activity of EPS

Antioxidant activity of the EPS was determined based on DPPH radical-scavenging, 2,2'-azino-bis (3-ethylbenzothiazoline-6-sulfonic acid) (ABTS)-scavenging, and ferric reducing antioxidant power (FRAP) activities. The EPS (500 μl) was mixed with DPPH radical solution (500 μl) and incubated in the dark at 25°C for 30 min. After centrifugation at 10,000 ×*g* for 1 min, absorbance was measured at 517 nm. The DPPH free radical-scavenging activity of the EPS was determined as previously described [21].

The ABTS solution was mixed with the same volume of EPS. After incubation at 37°C for 30 min, the absorbance at 734 nm was measured. The ABTS radical-scavenging activity was calculated as previously described [30].

The FRAP activity of EPS was determined as described previously [30]. After treating the EPS (100 μl) with FRAP reagent (900 μl) at 37°C for 30 min, the absorbance was measured at 593 nm. Ascorbic acid was the positive control.

### α-Glucosidase Inhibitory Activity of EPS and Kinetic Analysis

EPS (50 μl) was mixed with 50 μl α-glucosidase (1.0 U/ml; Sigma-Aldrich). After incubation at 37°C for 5 min, 50 μl of *p*-NPG was added as substrate, and the reaction proceeded at 37°C for 30 min. Kinetic parameters were determined using Michaelis–Menten kinetics [31, 32].

### Statistical Analysis

Using Duncan's multiple range test, statistical analysis based on at least three independent experiments was performed [33].

## Results and Discussion

### α-Glucosidase Inhibitory Activity of LAB

**Fig. 1:**
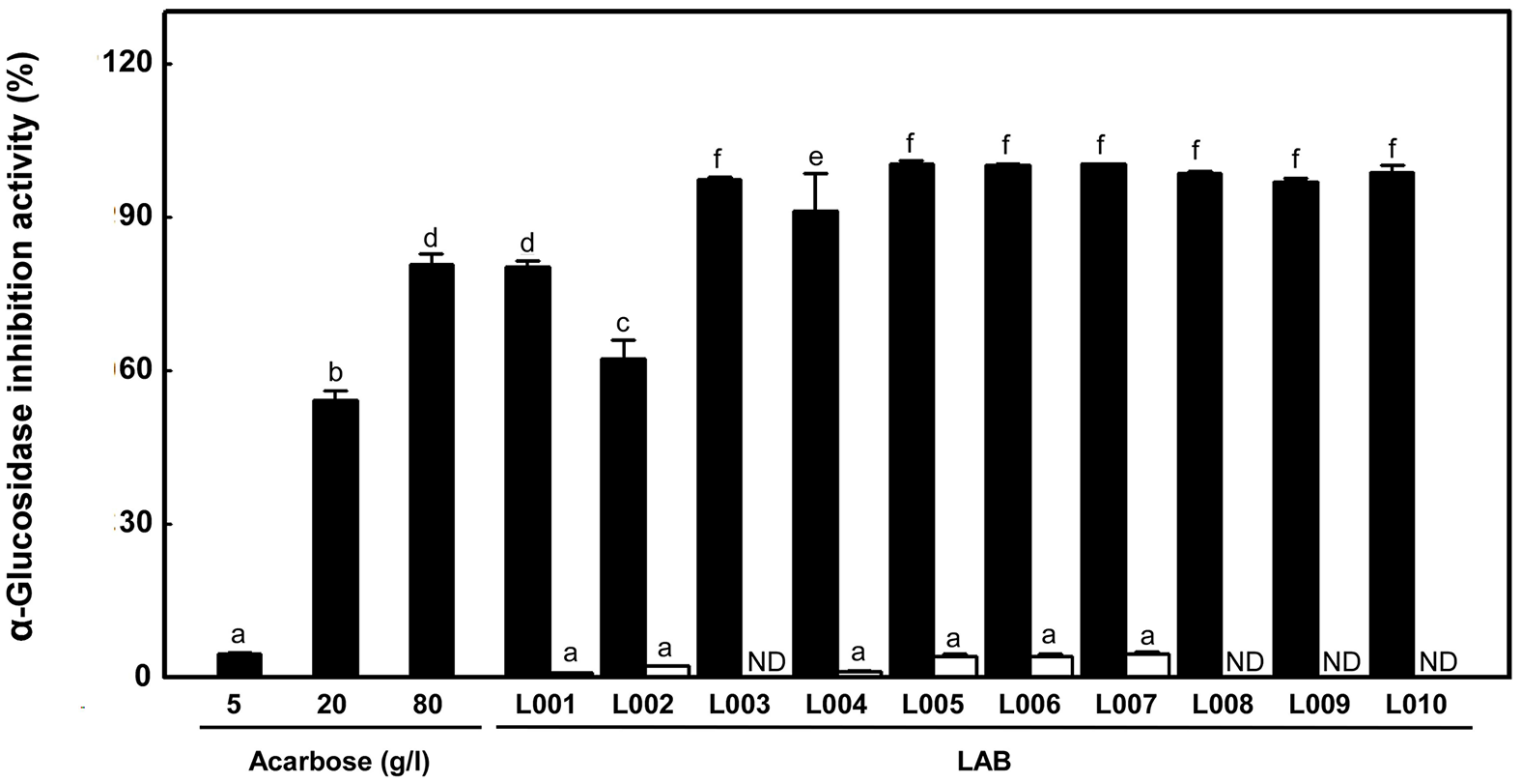
α-Glucosidase inhibitory activity of cell-free supernatant (■) and cell lysates (□) of LAB. All values are averages and standard errors determined from three independent experiments. Different letters indicate a significant difference between averages (*p* < 0.05). Acarbose was used as a control. ND: not detected.

The ten LAB exhibited α-glucosidase inhibitory activity (Fig. 1). The α-glucosidase inhibitory activity of the cell-free supernatant ranged from 62.30–100.29%, with strain L005 showing the highest activity (100.29 ± 0.80%) and L002 indicating the lowest activity (62.30 ± 3.53%). Cell lysates prepared from six strains showed α-glucosidase inhibitory activity. Among them, strain L007 showed the highest activity of 4.53 ± 0.45%. Nurhayati *et al*. [34] reported that the inhibitory activity of strain GN8 is 103.18 ± 3.96%, similar to the present study's activity. *Lactobacillus gasseri* 4M13 isolated from infant feces shows an inhibitory activity of >60% [35]. Chen *et al*. [14] reported that LAB cell lysates show approximately 0.81–4.53% inhibitory activities. Few studies have suggested that cell-free supernatants of LAB containing extracellular metabolites, such as EPS and inulin, inhibit α-glucosidase activity [14, 34].

### DPPH Radical-Scavenging Activity

**Fig. 2:**
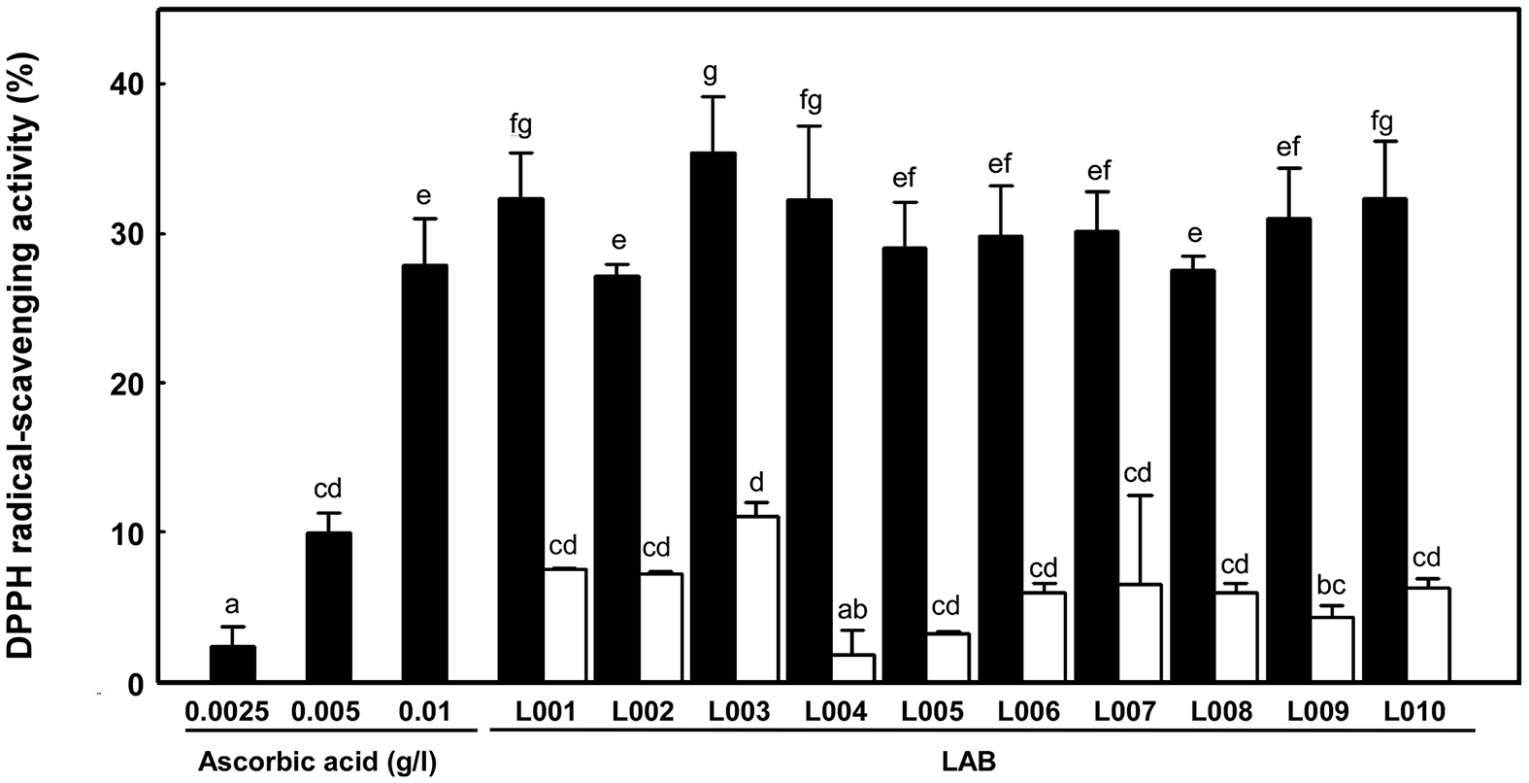
DPPH radical-scavenging activity of cell-free supernatant (■) and cell lysates (□) of LAB. All values are averages and standard errors determined from three independent experiments. Different letters on error bars indicate a significant difference between averages (*p* < 0.05). Ascorbic acid was used as a control.

The DPPH radical-scavenging activity was determined to evaluate the antioxidant activity of the LAB (Fig. 2). All LAB strains exhibited DPPH radical-scavenging activity ranging from 27.14–35.35% for the cell-free supernatant and 3.18–11.05% for the cell lysates. DPPH radical-scavenging activity of the cell-free supernatant was higher than those of the cell lysates in all tested strains. The L003 strain showed the highest DPPH radical-scavenging activities of 11.05 ± 0.89% and 35.35 ± 3.84% for cell lysates and cell-free supernatant, respectively, followed by the cell-free supernatants of L001 and L010, both of which showed approximately 32% DPPH radical-scavenging activity. *L. plantarum* B2 and *L. brevis* D7 have been reported to show radical-scavenging activities of 30.3 and 44.9%, respectively [36]. The authors suggested that the DPPH radical-scavenging activity is associated with EPS in the cell-free supernatant.

### Tolerance to Artificial Gastric Juice and Bile Juice Activity of LAB

**Fig. 3:**
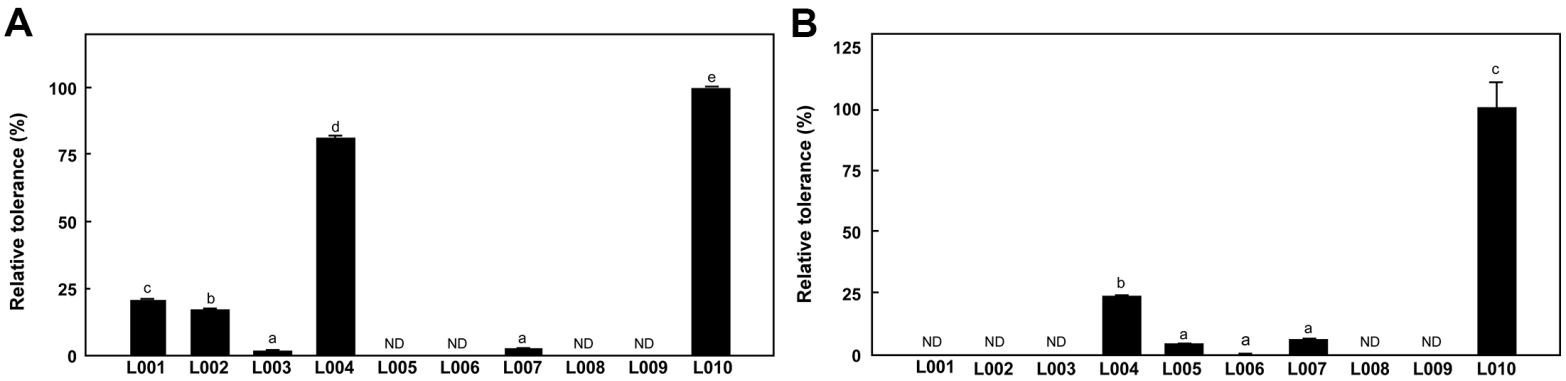
Tolerance of LAB in (A) artificial gastric and (B) bile juices. Relative tolerances were determined by comparing the OD_600_ of the strains with that of the L010 strain. All values are averages and standard errors determined from three independent experiments. Different letters on error bars indicate a significant difference between averages (*p* < 0.05). ND, not detected.

The effects of artificial gastric juice and bile juice on LAB cell viability are shown in Fig. 3. The viability of the L010 strain was the highest after exposure to artificial gastric juice, followed by that of L004. However, the L005, L006, L008, and L009 strains did not tolerate exposure to artificial gastric juice. Hassanzadzar *et al*. [24] reported that incubation at pH 2 and 3 decreases LAB viability. Among the ten LAB exposed to bile juice, five strains (L004, L005, L006, L007, and L010) showed tolerance, whereas the other five (L001, L002, L003, L008, and L009) did not. The tolerance of the L010 strain was considerably higher than that of the other strains.

### Adhesion of LAB to Caco-2 Cells

The adhesion rates of the LAB strains to Caco-2 cells ranged from 60.13–73.55% (Table 1). All strains could adhere to Caco-2 cells and exhibited higher adhesion rates than the LGG strain (41.22 ± 1.04%). The L010 strain showed the highest adhesion rate (73.55 ± 0.48%), approximately 1.8-fold higher than the LGG strain. *Lacticaseibacillus paracasei* subsp. *paracasei* E94, previously isolated from fermented olive, led to an adhesion rate (74%) similar to that of L010 [13].

### Antimicrobial Activity of LAB

**Fig. 4:**
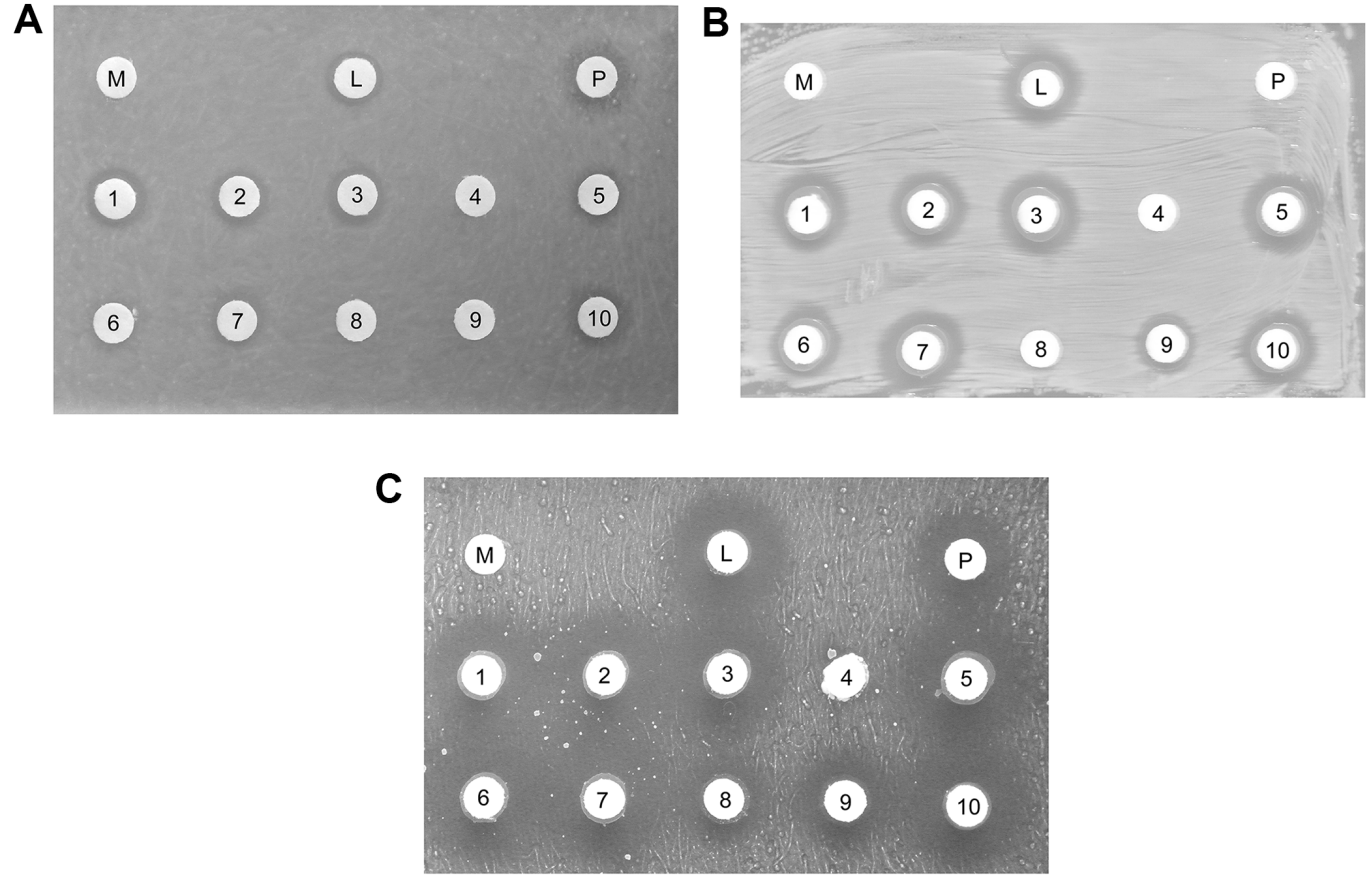
Antimicrobial activity of LAB against (A) *B. cereus*, (B) *E. coli* (B), and (C) *S. aureus*. M, MRS broth; L, LGG; P, Ampicillin (50 mg/l); 1, L001; 2, L002; 3, L003; 4, L004; 5, L005; 6, L006; 7, L007; 8, L008; 9, L009; 10, L010.

The antimicrobial activity against foodborne pathogens was tested for the LAB strains (Fig. 4). LAB inhibited the growth of all pathogens with inhibition zones ranging from 7.09–18.60 mm in diameter (Table 1). Among the ten LAB, seven strains showed antimicrobial activity against *B. cereus* with inhibition zones ranging from 7.09–8.54 mm in diameter. Nine strains inhibited the growth of *E. coli* with inhibition zones ranging from 9.10–12.15 mm in diameter. *S. aureus* was inhibited by nine LAB strains with inhibition zones ranging from 12.04–18.60 mm in diameter. The L001, 002, 003, 005, 007, and 010 strains showed antimicrobial activity against all three pathogens. The LGG strain, a positive control, also showed inhibitory activity against *B. cereus*, *E. coli*, and *S. aureus*. A recent study [37] reported that *L. plantarum* isolated from Ethiopian fermented food exhibits antimicrobial activity against *S. aureus* and *E. coli*. In agreement with the findings of this study, *L. brevis* KU15153, isolated from kimchi, shows distinct antimicrobial activity against *E. coli* and *S. aureus* [38].

### Identification of LAB

**Fig. 5:**
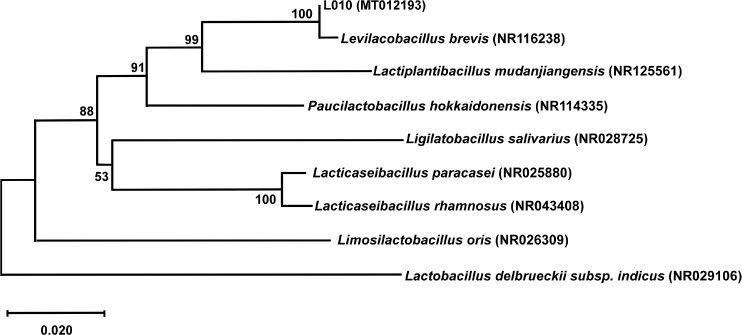
Phylogenetic relationships of *L. brevis* L010 with other strains. The relationship was constructed using the 16S rRNA gene sequence and the neighbor-joining method [47]. The GenBank accession numbers are shown in parentheses.

The L010 strain demonstrated antimicrobial activity and acid tolerance and was identified as *L. brevis*. To determine the molecular differences between the L010 strain and other strains, we constructed a 16S rRNA gene phylogenetic tree (Fig. 5). The L010 strain showed the highest similarity with an *L. brevis* (NR116238) strain at 100%, and it showed 99% similarity with *Lactiplantibacillus mudanjiangensis* (NR125561).

### Characteristics of Cell Growth

**Fig. 6:**
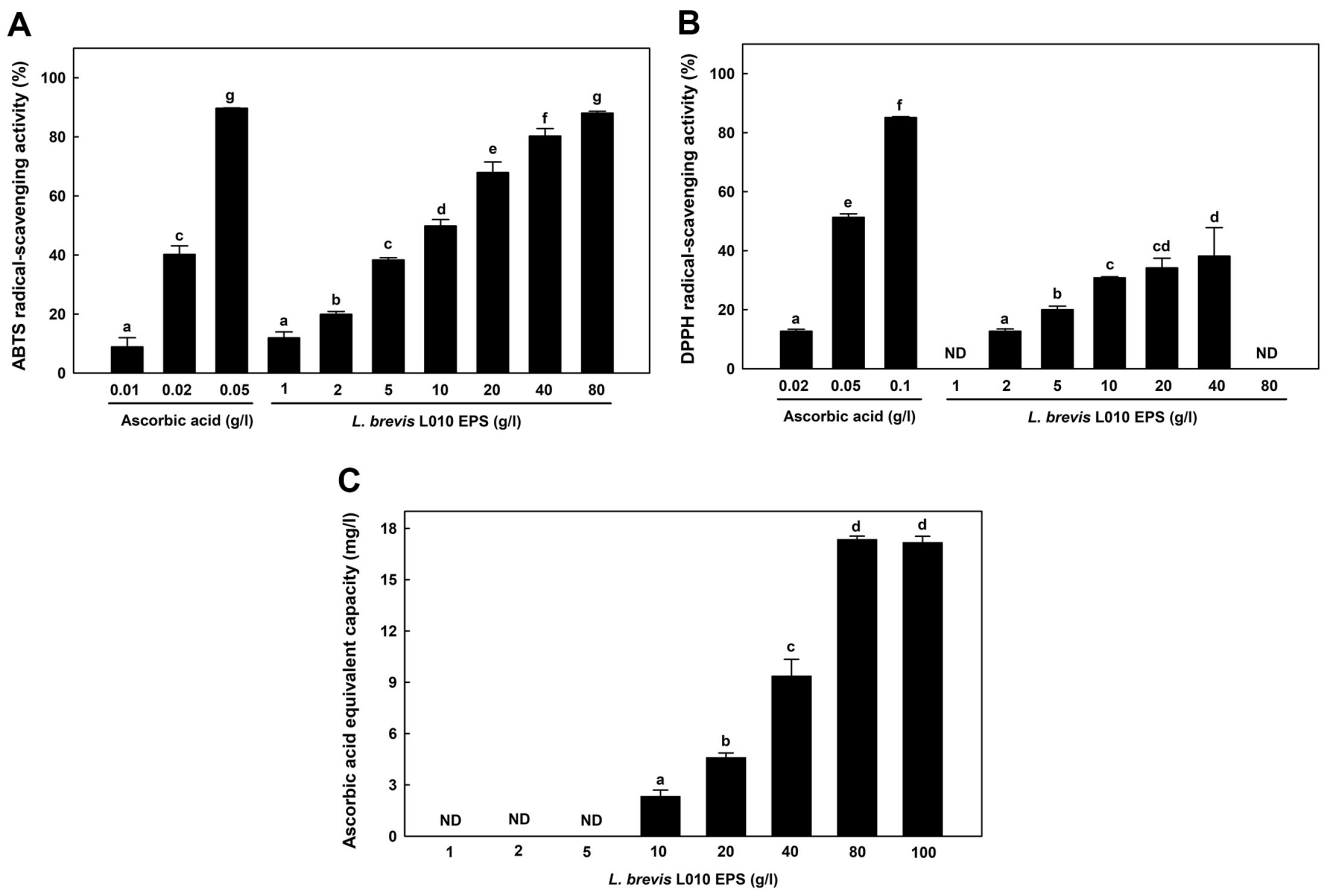
Antioxidant activity of EPS of *L. brevis* L010. (**A**) ABTS radical-scavenging activity, (**B**) DPPH radicalscavenging activity, and (**C**) FRAP activity. All values are averages and standard errors determined from at least three independent experiments. Different letters on error bars indicate a significant difference between averages (*p* < 0.05). Ascorbic acid was used as a control. ND, not detected.

The specific growth rate of *L. brevis* L010 ranged from 0.13–0.42 h^-1^ under various conditions (25–40°C, pH 5–7). Maximum cell growth rate of *L. brevis* L010 was observed at 30°C and pH 6 (Fig. 6); however, the strain did not grow at pH 4 and 8 (Fig. 6B). In general, *Lactobacillus* strains grow at 30–40°C and pH 5.5–6.2; however, certain strains can grow at 2–53°C and an initial pH of 4.5–6.5 [39]. A previous study showed that the specific growth rate of an *L. brevis* strain isolated from fruits is 0.34–0.46 h^-1^ at 30°C and pH 7, similar to the growth rate observed in the present study [40].

### Antioxidant Activity of EPS

**Fig. 7:**
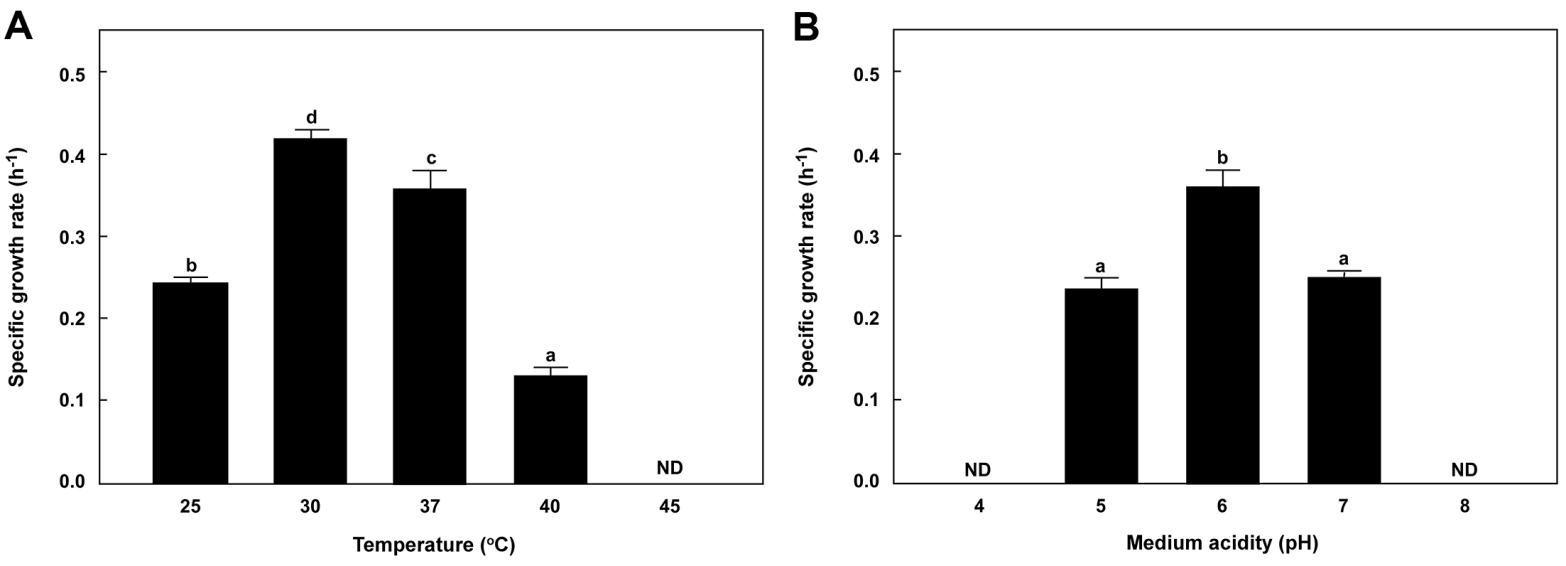
Effects of (A) temperature (at pH 6) and (B) medium acidity (at 30°C) on the specific growth rates of *L. brevis* L010. All values are averages and standard errors determined from three independent experiments. Different letters on error bars indicate a significant difference between averages (*p* < 0.05). ND: not detected.

The ABTS radical-scavenging activity of EPS was compared with that of ascorbic acid. EPS (80 g/l) showed an antioxidant activity (85%) equal to that of ascorbic acid (0.05 g/l) (Fig. 7A). The DPPH radical-scavenging activity of 40 g/l EPS was approximately 40% (Fig. 7B). Additionally, EPS (80 g/l) showed a FRAP activity of 17.56 ± 1.34 mg/l of ascorbic acid. No further increase in FRAP activity was observed at a concentration higher than 80 g/l (Fig. 7C). Recent studies reported that EPS produced by LAB exhibits antioxidant activity [41, 42]. The EPS of *L. plantarum* CNPC003 (8 g/l) exhibits 51.52 ± 1.10% DPPH radical-scavenging activity and 90.88 ± 0.80% ABTS radical-scavenging activity [43].

### Inhibition of α-Glucosidase Activity by EPS

The α-glucosidase activity was inhibited when 4 mM substrate was added; L010-derived EPS exhibited 7.05 and 23.30% inhibitory activity at 10 and 20 g/l, respectively (Table 2).

**Fig. 8:**
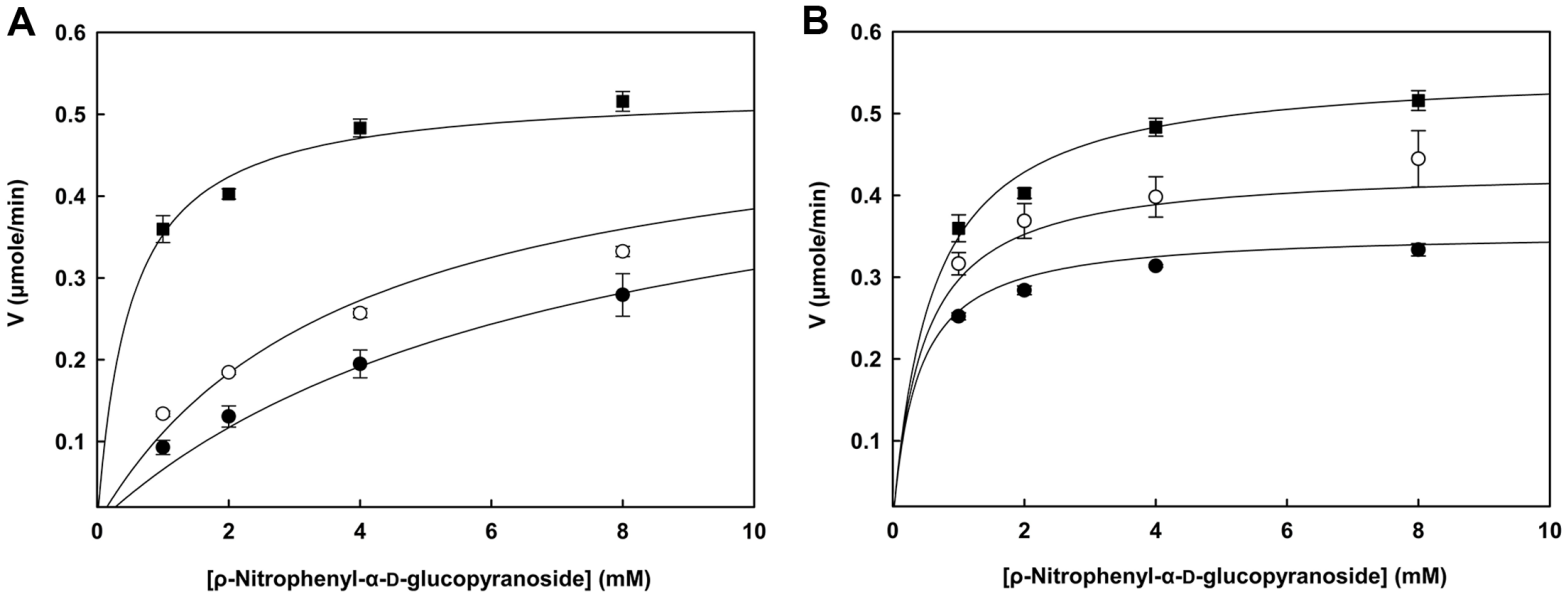
Inhibition of α-glucosidase activity by EPS of *L. brevis* L010. (**A**) Acarbose (○: 1 g/l, ●: 2 g/l, ■: water), (**B**) EPS of *L. brevis* L010 (○: 10 g/l, ●: 20 g/l, ■: water). Averages and standard errors were determined from three independent experiments.

To define the mode of inhibition, kinetics analysis was performed using optimal substrate concentrations (Fig. 8, Table 3). The results indicate that EPS competitively inhibited α-glucosidase, and the *K*_m_ values were 1.10 ± 0.03 and 2.87 ± 0.88 at 10 and 20 g/l, respectively (Table 3). Acarbose showed a mixed-type inhibition of α-glucosidase, and its *K*_m_ and *V*_max_ values increased as the compound concentration increased. Few recent studies similarly reported acarbose as a mixed-type inhibitor [44, 45]. Bajpai *et al*. [46] reported that crude EPS purified from Probio 65 shows inhibition rates of 7.05% at 10 g/l and 18.83% at 25 g/l in the presence of 5 mM substrate. The EPS was used in its crude form in the present study, and it is necessary to purify EPS via further purification and characterization.

In this study, *L. brevis* L010 isolated from jangajji showed a greater adhesion to Caco-2 cells than LGG. In addition, the strain displayed antimicrobial activity against foodborne pathogens, including *B. cereus*, *E. coli*, and *S. aureus*. It also showed greater tolerance to artificial gastric and bile juice than LGG. The EPS isolated from *L. brevis* L010 showed concentration-dependent antioxidant activity, and it competitively inhibited α-glucosidase. These results suggest that *L. brevis* L010 represents a probiotic candidate, and its EPS is attributed to the probiotic properties observed. The L010 strain was deposited in KCTC with Accession NO. KCTC14151BP.

## Figures and Tables

**Table 1:** Antimicrobial activity and adhesion rates to Caco-2 cells of LAB strains used in this study.

Strain	Inhibition size (mm)			Adhesion rate (%)	Isolation source
	*Bacillus cereus*	*Escherichia coli*	*Staphylococcus aureus*		
L001	8.54 ± 0.27^e^	11.10 ± 0.30^bc^	15.97 ± 0.08^c^	68.69 ± 0.45^g^	Makgeolli
L002	7.80 ± 0.29^cd^	11.24 ± 0.28^bcd^	16.99 ± 0.28^cde^	64.35 ± 0.24^d^	Makgeolli
L003	8.04 ± 0.24^d^	12.10 ± 0.16^f^	16.12 ± 0.46^c^	60.13 ± 0.34^b^	Jangajji
L004	ND	ND	ND	67.64 ± 0.30^f^	Kimchi
L005	7.09 ± 0.14^a^	11.82 ± 0.13^de^	16.56 ± 0.34^cd^	69.08 ± 0.55^g^	Kimchi
L006	ND	10.67 ± 0.21^b^	17.77 ± 0.28^ef^	66.56 ± 0.13^e^	Kimchi
L007	7.40 ± 0.12^ab^	12.15 ± 0.67^f^	18.60 ± 0.98^f^	66.55 ± 0.65^e^	Kimchi
L008	ND	ND	12.64 ± 0.33^a^	62.48 ± 0.03^c^	Kimchi
L009	ND	9.10 ± 0.03^a^	12.04 ± 0.55^a^	59.79 ± 0.11^b^	Jangajji
L010	7.48 ± 0.04^bc^	10.91 ± 0.23^bc^	17.22 ± 0.56^de^	73.55 ± 0.48^h^	Jangajji
LGG	8.04 ± 0.25^d^	11.48 ± 0.37^cd^	16.47 ± 0.43^b^	41.22 ± 1.04^a^	Human feces
Ampicillin (50 mg/l)	7.50 ± 0.19^bc^	ND	13.92 ± 0.43^cd^	-	-

*Averages and standard errors for inhibition size were determined from three independent experiments. Different letters indicate a significant difference between averages (*p* < 0.05). ND, not determined.

**Table 2:** α-Glucosidase inhibition activity of EPS from *L.brevis* L010 EPS.

	Inhibition activity (%)*
EPS (10 g/l)	7.05 ± 3.88^b^
EPS (20 g/l)	23.30 ± 3.50^c^
Acarbose (1 g/l)	34.81 ± 2.32^d^
Acarbose (2 g/l)	51.96 ± 5.49^e^
Water	0.0 ± 0.03^a^

*Averages and standard errors determined from three independent experiments are shown. Different letters indicate a significant difference between averages (*p* < 0.05).

**Table 3:** Kinetic constants of α-glucosidase reaction inhibited by EPS from *L. brevis* L010*.

	*V*_max_	K_m_	R^2^
EPS (10 g/l)	0.39 ± 0.01^a^	1.10 ± 0.03^a^	0.98
EPS (20 g/l)	0.39 ± 0.06^a^	2.87 ± 0.88^a^	0.93
Acarbose (1 g/l)	0.57 ± 0.06^b^	7.30 ± 1.55^b^	0.99
Acarbose (2 g/l)	0.95 ± 0.01^c^	21.94 ± 2.45^c^	0.99
Water	0.51 ± 0.03^b^	1.39 ± 0.21^a^	0.98

*Averages and standard errors determined from three independent experiments are shown. Different letters indicate a significant difference between averages (*p* < 0.05).
